# A Comprehensive Examination of the Role of Epigenetic Factors in Multiple Sclerosis

**DOI:** 10.3390/ijms25168921

**Published:** 2024-08-16

**Authors:** Ida Manna, Selene De Benedittis, Danilo Porro

**Affiliations:** 1 Institute of Bioimaging and Complex Biological Systems (IBSBC), National Research Council (CNR), Section of Catanzaro, 88100 Catanzaro, Italy; 2 Institute for Biomedical Research and Innovation (IRIB), National Research Council (CNR) Cosenza, 88100 Catanzaro, Italy; 3 Institute of Bioimaging and Complex Biological Systems (IBSBC), National Research Council (CNR), Segrate, 20054 Milan, Italy

**Keywords:** multiple sclerosis, epigenetic modifications, environmental factors, biomarkers

## Abstract

According to various research, the risk of multiple sclerosis (MS) is strongly influenced by genetic variations. Population, familial, and molecular studies provide strong empirical support for a polygenic pattern of inheritance, mainly due to relatively common allelic variants in the general population. The strongest MS susceptibility locus, which was unmistakably identified in tested populations, is the major histocompatibility complex on chromosome 6p21.3. However, the effect of a given predisposing variant remains modest, so there is the possibility that multiple gene–gene and/or gene–environment interactions could significantly increase the contribution of specific variants to the overall genetic risk. Furthermore, as is known, susceptibility genes can be subject to epigenetic modifications, which greatly increase the complexity of MS heritability. Investigating epigenetic and environmental factors can provide new opportunities for the molecular basis of the MS, which shows complicated pathogenesis. Although studies of epigenetic changes in MS only began in the last decade, a growing body of literature suggests that these may be involved in the development of MS. Here, we summarize recent studies regarding epigenetic changes related to MS initiation and progression. Furthermore, we discuss how current studies address important clinical questions and how future studies could be used in clinical practice.

## 1. Introduction

**Figure 1:**
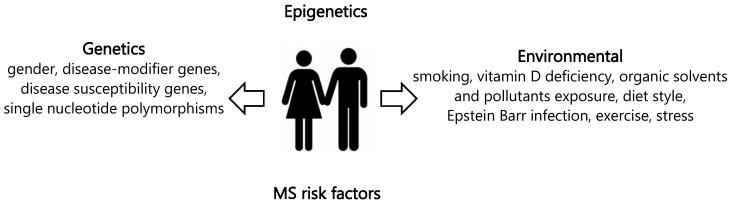
Risk factors of multiple sclerosis.

Multiple sclerosis (MS) is a disorder of the central nervous system (CNS), characterized by both inflammatory and neurodegenerative features. Autoreactive CD4 T cells are thought to be the primary cause of MS pathogenesis. Activated in the peripheral, these cells cross the blood–brain barrier (BBB) to enter the CNS along with activated myeloid cells and B lymphocytes [1]. Following their reactivation in the CNS, invading cells and immune cells that spread in the brain create an inflammatory environment that causes axon demyelination and neuronal death. Demyelinating lesions, which affects the myelin sheath surrounding the axon, causing axonal degeneration and neuronal loss in the brain, are a hallmark of MS [2]. By destroying the myelin sheath, this disease compromises the transmission of nerve impulses which are followed by neurological episodes that compromise their function and cause disability [3]. MS can present in four different clinical phenotypes which are important not only to predict the course of the disease, but also to decide on the appropriate therapeutic approach: (1) relapsing–remitting multiple sclerosis (RR-MS); (2) primary–progressive multiple sclerosis (PP-MS); (3) secondary–progressive multiple sclerosis (SP-MS); and (4) progressive–relapsing multiple sclerosis (PR-MS). Each form of MS has a different symptom pattern and course of the disease [4]. Neurological dysfunctional episodes may occur in RR-MS patients with or without residual damage. Of the several patterns, RR-MS is the most common clinical type of the disease. It is characterized by repeated episodes of remission brought on by the immune system shutting down and relapse brought on by autoimmune aggressiveness [5,6]. In total, 15% to 30% of patients with RRMS will proceed to secondary progressive MS, which causes progressive disability. Last but not least, 15% of people have primary progressive MS, which impairs neurologic function from the moment symptoms arise without relapses or remissions [7]. Several clinical investigations, including clinical examination, magnetic resonance imaging, measurement of cerebrospinal fluid, and electrophysiology, are crucial for the diagnosis and monitoring of MS. Although the precise etiology of MS is still unknown, it is thought to be caused by an aberrant immune response to one or more myelin antigens that appear in genetically vulnerable people following exposure to an as-yet-unidentified causative agent. The genetic predisposition to MS is polygenic. The major histocompatibility complex (MHC), also called the human leukocyte antigen (*HLA*) system, was the first to be linked to the disease. More than 200 polymorphic variations associated with MS were found using genome-wide association studies, with the haplotype HLA-DRB1*15:01 having the most impact. Although MS risk loci confer only modest individual effects, they do converge into pathways that are significant for T cell and adaptive immunity activities. In addition, cigarette smoking, mononucleosis, the pathogenic Epstein–Barr virus (EBV), and low vitamin D levels were all repeatedly linked to an increased risk of MS [8]. Furthermore, compared to the effects of each genetic risk factor alone, smoking was demonstrated to interact with HLA-DRB1*15:01, *HLA-A2*, and *NAT1*, significantly increasing the probability of developing MS [9]. Hypovitaminosis D was proposed as a potential risk factor for MS based on study findings from the past few decades about the relationship between MS and vitamin D status. MS susceptibility was linked to certain gene variations encoding proteins involved in vitamin D metabolism, transport, and activity, which are responsible for changes in vitamin D status [10]. The first study conducted to evaluate the possible influence of different SNPs in vitamin D-related genes on the severity of MS did not highlight any association between the SNPs investigated and the progression of MS [11]. Other case-control studies did not show any association between selected SNPs and susceptibility to MS [12,13,14]. Overall, these studies do not offer sufficient results to say whether vitamin D-related genetic variants influence MS risk or not. However, several statistical data suggest that the contribution of all MS-associated variations discovered to date may only account for up to 48% of the heritability of the disease, highlighting the possible role of interactions between environmental and genetic factors [15]. In addition to environmental and genetic factors, epigenetic factors also influence the risk of onset and can mediate part of the risk associated with environmental factors by altering gene expression. Epigenetics also might be the link between environmental variables and MS susceptibility (Figure 1).

To further understand the molecular mechanisms of MS, epigenetic modifications should also be investigated. The field of epigenetics investigates how a person’s age and exposure to environmental elements, such as chemicals and physical hazards, food, and physical exercise, can affect their lifetime and vary depending on the type of cell or tissue they are in.

**Figure 2:**
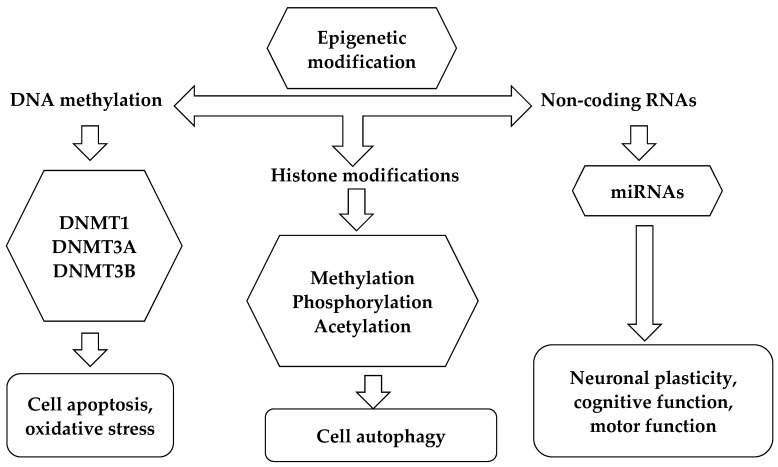
Epigenetic mechanisms.

Epigenetic marks are heritable, independent of changes in DNA sequence, and modulate gene expression. Indeed, these changes can trigger or influence critical cellular responses that may be crucial in determining disease phenotype. Various environmental factors, including food, smoking, and stressful conditions, can impact the epigenome’s plasticity and play a role in epigenetic regulation. A schematic diagram of the different types of epigenetic mechanisms is illustrated in Figure 2.

Briefly, DNA methylation consists of the addition of a methyl group (-CH3) to a base, such as methylation at the carbon-5 level of cytosine, which constitutes almost all methylation of eukaryotic DNA. 5-methyl-cytosine (5mC) is found almost exclusively in the cytosine-phosphate-guanine (CpG) dinucleotide in the coding region of genes [16]. The main function of DNA methylation is associated with the inactivation of gene expression and could also contribute to transcriptional regulation by influencing chromatin remodeling [17], and alternative splicing [18]. The process is produced by a family of enzymes called DNA-methyl-transferase (DNMT). In the genome, almost 80% of CpG sequences are methylated [19]. The transfer of a methyl group is catalyzed by three DNA methyltransferases: DNMT1, DNMT3A, and DNMT3B. While DNMT3a and DNMT3b, together with their coactivator DNMT3L, are engaged in de novo DNA methylation and in DNA methylation maintenance, DNMT1 is in charge of methylating hemimethylated DNA and thereby maintaining DNA methylation [20]. More recently, a fourth DNA methyltransferase enzyme, called DNMT3C, was discovered by Barau et al., in mouse germ cells. This enzyme is responsible for methylating the promoters of evolutionarily young retrotransposons and its activity is required for mouse fertility. In addition to expanding knowledge of the mechanisms involved in the epigenetic regulation of retrotransposons, the identification of DNMT3C highlights the plasticity of the DNA methylation system [21]. Methylation of cytosine residues causes gene silencing possibly by blocking the binding of transcription factors to promoters; however, in some cases, methylated DNA motifs can also be specifically recognized by transcription activators [22]. 

Another well-known fundamental epigenetic mechanism, which controls gene expression without altering the genetic material, is the modifications of the histone tails. When we talk about histone modification we refer to the addition of chemical groups to histones, the proteins around which the long strand of DNA wraps itself so that it is “packaged” and contained in the nucleus of the cell [23]. There are five types of histones in eukaryotic cells: H1, H2A, H2B, H3, and H4. Two copies of histones H2A, H2B, H3, and H4 form a protein complex called a histone octamer, around which the double strand of DNA wraps. The fifth type of histone, H1, stabilizes the binding of DNA to histones in chromatin. Histones can undergo various modifications, among which the most common are acetylation (addition of an acetyl group) and methylation, which lead to variations in the structure of chromatin, making it more or less accessible to RNA polymerase, resulting in activation or repression of genes associated with the modified histone [24]. The best-understood histone modification is acetylation and deacetylation at the lysine residues that are carried out by histone acetyltransferases (HAT) and histone deacetylases (HDAC), respectively. Acetylation of histones is usually associated with upregulated transcriptional activity of the associated gene, whereas deacetylation of histones contributes to transcriptional silencing. In fact, modifications such as acetylation and methylation of the tails, in specific amino acid residues of specific histones, have the effect of activating or inhibiting transcription, according to a real histone code [25]. Indeed, the functional consequences of these changes on transcription depend on the type of change and the location of the targeted amino acid residue within the histone tail. 

An additional mechanism of epigenetic control can also be achieved at the post-transcriptional level by small non-coding RNAs (ncRNAs). Among ncRNAs, microRNAs (miRNAs) are small endogenous single-stranded molecules that modulate messenger RNA activity. The role of miRNAs is to negatively regulate gene expression through binding to the target mRNA to cause transcriptional repression and/or mRNA degradation without modifying the gene sequence [26]. It is interesting to note that miRNAs typically change the activity of pathways since they can regulate the synthesis of multiple downstream targets not only a single mRNA [27]. miRNAs were shown to regulate more than 60% of protein-coding genes. Since this complex “network” is normally very finely regulated, alteration in microRNA expression can lead to the development of various diseases [28]. The abundance of miRNAs in the CNS can impact a variety of cellular activities, including differentiation, growth, proliferation, plasticity, and development [29]. miRNAs can be considered epigenetic components involved in immune regulation and MS pathogenesis due to their ability to alter gene expression, and their role in physiology and numerous pathological processes. Immune cells also exhibit high expression of miRNAs. Furthermore, miRNAs are detectable in a wide variety of body fluids [30], and consequently, peripheral circulating miRNAs may be employed as biomarkers for MS diagnosis, MS subtype differentiation, and MS prognosis prediction. 

In the development of MS, the interaction between hereditary determinants and cell-specific epigenetic alterations may be extremely important. Epigenome modifications caused by environmental risk factors for MS create a connection between environmental exposure and changes in gene expression [31]. An increasing amount of research points to the importance of dietary nutrients in inducing epigenetic modifications for health and disease prevention, particularly for neurodegenerative illnesses. Folic acid, vitamin B12, and dietary polyphenols were demonstrated in numerous studies to interact with epigenetic pathways and ultimately gene regulation [32]. Epigenetic changes were also implicated in developmental myelination and remyelination [33]. The complex genetic profile of MS and its epidemiological traits led us to hypothesize that the balance between the genetic and epigenetic components may be crucial for disease susceptibility, onset, and progression. Finally, studying the epigenome in MS provides valuable insights into the pathophysiology of disease progression and repair. Although the mechanisms involved in the development of MS are not yet fully defined, understanding the salient characteristics of the evolution of lesions in the different areas and phases of the disease is the basis for the development of increasingly targeted and effective therapies. For this purpose, considering the cellular specificity of epigenetic changes, in this review, we discuss the most relevant findings to date in different tissues of MS patients. Finally, we examine their potential implications for the future application of epigenetic advances in the field of MS.

## 2. DNA Methylation: Contribution in Patients with MS

DNA methylation is a crucial epigenetic process that controls gene expression in a dynamic and reversible manner. The transcription of genes is typically activated or repressed depending on whether a methyl group is present or absent on CpG dinucleotides. DNA methylation is modulated by environmental variables, including nutrition, stress, and exposure to the environment [34]. Recent studies connected neurological illnesses to changes in DNA methylation [35]. These epigenetic modifications function as simple tools for controlling the expression of the vital gene networks that mediate physiological processes. The specific pathophysiological mechanisms mediating the relationship between environmental risk factors and MS susceptibility remain unknown, and DNA methylation may provide information on these mechanisms [36]. This epigenetic pathway contributes to various MS pathophysiological processes, such as demyelination and remyelination, inflammatory response, and the collapse of the BBB [37]. It is also linked to chronic inflammatory states. DNA methylation appears to play a major role in the epigenetics of MS, as aberrant methylation in the promoter regions of the genome may be the cause of many processes involved in the beginning and development of MS. Since DNA methylation is involved in many neurodegenerative diseases, modifying DNA methylation may be a useful therapeutic approach in treating these conditions. It was described that methylation of CpG dinucleotides in gene promoter regions could be responsible for the immune properties of MS, with the dominance of T helper 1 cell immunity, associated with the cytokine interferon-γ, compared to T helper 2 cell immunity. The promoter region of IFN-γ within T helper cells showed aberrant patterns of DNA methylation, which could be associated with the predominance of TH1 immunity over TH2 immunity in MS [38]. The BBB, which is thought to be composed of endothelial cells and intercellular adhesion molecules, is known to allow the passage of self-reactive proinflammatory cells, a pathogenic characteristic of multiple sclerosis. It was demonstrated that in RR-MS patients, a hypermethylated E-cadherin (*CDH1*) profile can increase blood–brain barrier permeability, allowing lymphocyte infiltration into the brain and, ultimately, the progression of the disease [39]. Referring to the *ICAM* family, namely *ICAM-1*, which is essential for T cell proliferation, Ligget et al., note that cell-free plasma DNA of RR-MS patients have an *ICAM1* hypermethylation pattern in response to clinical remission, and the observed differences mostly correlated with the disease process. Significant differences in concentration and the methylation patterns of cell-free plasma DNA were detected in all three comparisons: RRMS patients in remission versus healthy controls; RRMS patients in exacerbation versus healthy controls; and RRMS patients in exacerbation versus those in remission [40]. These results may have important therapeutic ramifications since methylation patterns that distinguish patients from healthy individuals may serve as biomarkers for the identification and diagnosis of disease. Moreover, a predictive biomarker for exacerbation may result from variations in the methylation patterns of patients in remission and/or exacerbation. Remyelination failure in the advanced stage of MS is ultimately caused by the impaired differentiation of oligodendrocyte precursor cells (OPCs). Using postmortem brain tissue, genome-wide DNA methylation and transcriptional patterns were examined between matched normal-appearing white matter (NAWM) and chronically demyelinated MS lesions. Using pyrosequencing, it was confirmed that DNA methylation variations in laser-captured OPCs were exclusive to cell type and negatively linked with mRNA expression of the respective genes. Through the application of epigenetic editing to alter the DNA methylation status of particular CpGs within the promoter region of the myelin basic protein gene (*MBP*), it was demonstrated that the CRISPR–dCas9–DNMT3a/TET1 system may be used to bidirectionally influence myelination and cellular differentiation in vitro [41].

Recently, Tiane et al. [41,42] looked into the possibility that a blood-borne biomarker for demyelination may be found in the blood methylomic profile of myelin-related genes, and that this profile might be systemically changed in advancing MS stages. Their preliminary research showed that patients with progressive MS differed from controls in the blood methylation state of key myelin-related genes: *MBP*, myelin-associated glycoprotein (*MAG*), contactin 2 (*CNTN2*), brain-enriched myelin-associated protein 1 (*BCAS1*), partitioning defective 3 homolog (*PARD3*). Among the five genes, three (*MBP*, *MAG*, and *CNTN2*), in particular, displayed a methylation pattern that differed significantly between the control and MS samples. It is interesting to note that these genes’ methylation profiles matched the pattern found in the central nervous system, indicating systemic hypermethylation of these genes in MS patients with progressing disease. This suggests that a panel of peripheral markers, useful for assessing demyelination in the course of MS disease, could employ the DNA methylation profile of these genes. Although, these findings were not confirmed in further independent patient cohorts [42]. More longitudinal research on bigger cohorts of diverse ethnic backgrounds, including both patient and control groups, would be necessary to fully test the findings of these investigations.

Research on how DNA methylation affects MS in rodent models, especially in mice with experimental autoimmune encephalomyelitis (EAE), demonstrated significant potential for application to human MS patients [43]. The majority of attempts to identify variations in DNA methylation throughout the genome fall into two categories: genome-wide methylation investigations and candidate gene approaches, which involve selecting specific genetic loci and examining their DNA methylation patterns.

Additionally, combinations of these methods can be used [44]. To date, several researchers linked the methylation status of specific genes to MS. In particular, Kumagai et al. observed of the promoter of tyrosine phosphatase (*SHP-1*) a negative regulator of proinflammatory signaling in peripheral blood mononuclear cells (PBMCs) of MS patients compared to healthy controls. Hypomethylation results in decreased *SHP-1* expression and thus increased lymphocyte-mediated inflammatory activity [45]. In another work, Janson et al., analyzed CD4+ T cells in a cohort of RR-MS patients and observed that the regulatory T cell transcription factor *FOXP3* was demethylated in these patients. Demethylation of *FOXP3* stimulates regulatory T cells and Th17 cells while inhibiting the development of Th1 and Th2 cells. The proper balance of these cells is linked to DNA methylation [46]. In addition, hypomethylation of the promoter of the gene encoding *IL-17A*, a proinflammatory cytokine, mainly secreted by activated T cells, was reported [47]. In the work of Mastronardi et al., during white matter demyelination in MS patients, the authors observed demethylation of the peptidyl arginine deiminase 2 (*PAD-2*) promoter and thus *PAD-2* overexpression in the brain. *PAD-2* leads to the loss of stability of *MBP* due to the enzymatic conversion of arginine to citrulline. This process leads the *MBP* protein to act as an antigen for T cells. The authors observed a 25% reduction in white matter *PAD-2* promoter methylation in patients compared to healthy controls. Of note, these changes were not seen in patients with other neurological diseases such as Alzheimer’s disease, Parkinson’s disease, and Huntington’s disease [48].

Literature data report genome-wide DNA methylation profiling in MS patients, brain tissues, whole blood, and immune cells. Because CD4+ and CD8+ immune cells are the most prevalent cell populations, with a well-documented role in the genesis and progression of MS, they were examined more frequently than other immune cell types. Although the precise role of DNA methylation in MS is not fully understood, several studies reported differentially methylated regions in both immune cells and brain tissue collected from both MS patients and healthy controls. The ability to identify the neurotoxic and pro-inflammatory mechanisms causing neuronal vulnerability in MS progression made the study of glial cell populations particularly interesting [49]. This could lead to the eventual restoration of brain repair mechanisms that support neuroprotection and remyelination. Oligodendrocytes, which are more prevalent in the white matter (WM) than the grey matter (GM) due to their capacity to produce myelin, give neurons structural and trophic support by regulating the myelination of axons [50]. Numerous minor differences between the bulk NAWM of MS patients and controls were found by previous case-control methylome studies of brain tissue. In the work of Huynh et al., genome-wide RNA-sequencing analysis helped to further identify the transcriptional effects of differential DNA methylation, and it was confirmed in two separate cohorts of brain samples from patients with multiple sclerosis and healthy controls. In brains affected by MS, genes controlling oligodendrocyte survival, such as *BCL2L2* and *NDRG1*, were expressed at lower levels and showed hypermethylation compared to controls. Conversely, increased levels of hypomethylation and expression were seen in genes associated with proteolytic processing, such as *LGMN* and *CTSZ*. According to their findings, there may be a trend toward hypomethylation in genes linked to immune-related activity and hypermethylation in genes involved in oligodendrocyte function [51]. Chomyk et al. looked at whether demyelination in the MS hippocampal region affected DNA methylation. When MS patients’ demyelinated and myelinated hippocampi were compared, abnormal epigenetic alterations linked to the dysregulation of a few select genes were found. The demyelinated MS hippocampal region exhibits hyper-methylation in 10 genes and hypo-methylation in 6 upstream sequences according to comparative methylation profiling [52]. Recent research of Kular et al., using DNA methylation analysis of neuronal nuclei, revealed functionally significant alterations, such as decreased activity of the transcription factor *CREB*, linked to neuro-axonal dysfunction in MS patients relative to controls. The two cohorts’ pathway studies revealed dysregulation of genes related to synaptic plasticity and axonal guidance, and a meta-analysis confirmed that the most significantly enriched route underlying these processes is CREB signaling [53]. Recently, using the Illumina Infinium MethylationEPIC BeadChip, Kular et al., profiled DNA methylation in the nuclei of non-neuronal cells isolated from 38 post-mortem NAWM specimens of MS patients (n = 8) in comparison to the white matter of control individuals (n = 14) in order to capture relevant molecular changes underlying MS neuropathology. They found 1226 significantly differentiably methylated sites (DMPs) between MS patients and controls (genome-wide adjusted *p*-value < 0.05). The modified DMP-genes’ functional annotation revealed changes in metabolic processes, cytoskeleton dynamics, cellular motility, synaptic support, neuroinflammation, and signaling, including Wnt and TGF-β pathways. Their results clearly imply that even in the absence of lesional damage, NAWM glial cells are significantly changed, showing a multicellular response to widespread inflammation [54]. 

Several studies showed an aberrant DNA methylation profile in RR and progressive MS forms [55]. Kiselev et al., to identify differential methylation profiles of CpG sites in the DNA of CD14+ monocytes from patients with primary progressive MS, a genome-wide analysis of DNA methylation in patients compared to healthy subjects was conducted. The study highlighted 169 differentially methylated positions (DMPs), 90.5% of which were hypermethylated in PPMS patients [56]. In recent years, many research teams examined the impact of aberrant DNA methylation on MS characteristics, and they reached a variety of conclusions. However, to date, the results of published genome-wide DNA methylation studies are not consistent and may be associated with the choice of non-uniform criteria, such as heterogeneity of the groups studied, sample size, different methodologies, and statistical strategies used. In addition, further research indicates that sample storage may affect DNA methylation patterns selectively, which could make epigenetic analysis less accurate. As a result, while choosing samples for biomarker research, sample storage period should be taken into account. Indeed, regarding the selection of the group of patients with MS, not only naive patients are included in the various studies, but also patients who received various immunomodulatory drugs before and/or during blood sampling, when it is well known that drug therapy affects the level of methylation in blood cells [57]. Comparing the DNA methylation profiles of other cell groups from the same individuals may help to address some of these issues and provide more accurate information on the pathophysiology of MS. 

About it, little research the literature conducted on DNA methylation analysis takes into account various immune cell populations obtained from the same MS patients. Ewing et al., investigated DNA methylation changes in four immune cell types, i.e., CD4+ and CD8+ T cells, CD14+ monocytes, and CD19+ B cells, in a cohort of patients with RRMS and SPMS and healthy controls. They found 1511, 666, and 30 significant differentially methylated positions (DMPs) in CD19+, CD14+, and CD8+ cells, respectively, between RRMS, SPMS, and HC individuals, with two significant DMRs mapping to *HLA-DRB1* and *SLFN12* genes [58]. Recently, Kiselev et al., considering cell populations key in triggering pathological processes underlying the development of MS, performed pairwise DNA methylation profiling in CD4+ T lymphocytes and CD14+ monocytes. They observed significant changes in the DNA methylation profiles among these cell populations, and they highlighted that in CD4+ cells of MS patients, most of the DMPs were hypomethylated, while in CD14+ cells they were hypermethylated. However, they observed that approximately 20% of the identified DMPs were shared between the two cell populations from treatment-naïve RR-MS patients and healthy subjects [59]. Although these findings suggest that differential DNA methylation in immune cells could contribute to MS, further studies are needed to validate these findings and especially to understand their functional significance. In genome-wide association studies, the strongest association between MS and differential DNA methylation occurs at the *HLA-DRB* locus. Despite the HLA genes being among the highest genetic predictors of MS risk for more than 40 years, the precise causative genes and the processes by which they affect MS susceptibility remain unknown. 

Graves et al., provide the findings of a genome-wide link between CD4+ cell methylation levels and susceptibility to MS. The significant differential methylation signal at CpG sites throughout the MHC complex, particularly the hypomethylation of eight closely clustered sites in *HLA-DRB1*, was the study’s most notable observation. The hypomethylation at *DRB1* may be a key epigenetic locus for MS, and this is the first instance of aberrant methylation at HLA being linked to MS [60]. Recently, to understand the biological effects of inheritance of MS risk alleles and show that DNA methylation influences risk of developing MS, Kular et al. examined DNA methylation in MS patients in the context of genetic diversity and gene expression. In particular, the protective HLA variant (rs9267649) and the strongest MS risk variant (HLA-DRB1*1501) have an impact on *HLA-DRB1* expression and the risk of MS through DNA methylation. Their findings highlight that the region containing exon 2 of DRB1*15:01 is regulated by DNA methylation, and hypomethylated DRB1*15:01 has a capacity to promote increased gene expression. Therefore, DRB1*1501’s hypomethylation and predominant expression may be a possible mechanism by which MS risk is conferred [61]. More recently, Xavier et al., confirmed prior findings that DNA methylation acts as a mediator of the HLA-DRB1*1501 allele and provided evidence that this occurs early in disease pathology rather than as a result of treatment or long-standing disease by using a large dataset of MS cases early in their diagnosis with age/sex/location-matched controls. They examined the relationship between methylation and expression levels of *HLA-DRB1* in monocytes, CD19+ B cells, and CD4+ T cells, and found a strong negative correlation between gene expression and methylation at *DMR-2* located in exon 2 of *HLA-DRB1*. These data indicate that hypomethylation at this locus was associated with increased gene expression in these immune cell subtypes [62]. Furthermore, in their study they examined whole blood DNA methylation profiles of a large group of MS subjects compared to those of healthy control subjects, thus highlighting that methylation differences in patients occur independently of genetic risk loci, subsequently proving that they differentiated the disease more strongly than known genetic risk loci. Table 1 lists the studies discussed in the manuscript.

In conclusion, the genome-wide DNA methylation changes in whole blood and isolated cell types were the subject of numerous studies. Even though results are inconsistent and limited by heterogeneous cell populations, disease heterogeneity, the use of a wide variety of disease-modifying therapies (DMTs), or small sample sizes, there is no doubt that DNA methylation is influenced by genotype and environment, making it an important molecular interface for researching disease etiology and progression. 

## 3. Post-Translational Histone Modifications: Contribution to MS

Post-translational modifications (PTMs), which occur when proteins are translated into the cytoplasm, are crucial for the folding and functionality of proteins [63]. Among the most common histone changes are histone acetylation/deacetylation, and histone methylation. Two opposing enzymatic activities regulate histone acetylation: histone acetyl transferase o HAT, and histone deacetylase or HDAC. HATs add acetyl groups on lysine residues, while HDACs remove them. 

According to growing data, epigenetic changes may hold the key to understanding the partial heredity of MS risk. Indeed, epigenetic pathways are thought to mediate the response to a variety of environmental factors, including geographic location, Epstein–Barr virus (EBV) infection, smoking, and vitamin D deficiency, which are known to affect the development of the disease [64,65,66,67]. It is well known that *HLA-DR* expression was shown to be suppressed from *HDAC1*. The connection between inflammation and neurodegeneration and *HDAC/HAT* imbalance may have an effect on MS mechanisms. HDAC mRNAs were previously demonstrated to be higher in immune cells that were activated, indicating their possible uses as therapeutic targets or activation markers [68]. This suggests that the MS susceptibility loci are related to histone regulation. In the work of Pedre et al. the authors observe increased levels of histone H3 acetylation in chronic lesions of MS patients compared to healthy controls. Notably, they point out that in oligodentroglia cells, changes were associated with higher transcript levels of inhibitors of oligodendrocyte differentiation. Interestingly, deacetylation was evident in early MS lesions, unlike in chronic lesions. This observation highlights the important role of the acetylation/deacetylation balance in the demyelinating and remyelinating processes in the early stages of the disease [69]. Furthermore, regarding changes in histone H3 methylation, the latter linked to mitochondrial alterations in MS. Singhal et al., in a study, measured the concentrations of methionine metabolites, histone H3 methylation patterns, and markers of mitochondrial respiration in gray matter from postmortem MS and control cortical samples. They found decreases in both respiratory markers and concentrations of methionine metabolites in the gray matter of MS [70]. In another work, the study group of Singhal et al., demonstrated that methyl donor betaine is reduced in MS and is linked to decreases in histone H3 trimethylation (H3K4me3). They also observed that betaine increases histone methyltransferase activity by activating chromatin-bound betaine homocysteine S-methyltransferase (BHMT) [71]. In a recent study, Singhal et al. linked histone PTMs to communication between energetically impaired neurons and oligodendrocytes to promote remyelination in MS. They observed reduced levels of the metabolite N-acetylaspartate (NAA) in MS patients that correlated with reduced levels of the myelin component in apparently NAWM [72]. This finding is in agreement with previous work related to neuronal metabolic dysfunction, linked to a marked decrease in NAA metabolite in postmortem multiple sclerosis cortex, that precedes neuronal atrophy and can be reversed in disease remission conditions. Reduced NAA and neuronal mitochondrial dysfunction in MS may influence the epigenetic changes of histones, which in turn mediates regulatory signals in oligodendrocytes. In the end, altered myelin lipid content and oligodendrocyte gene expression may make axons more susceptible to demyelination as a result of decreased NAA synthesis in neurons and catabolism in oligodendrocytes [73]. 

In a more recent work, Sternbach et al. investigated the role of the BHMT-betaine methylation in oligodendrocytes. They determined the presence of the BHMT enzyme in oligodendrocytes and the role of the BHMT–betaine pathway in chromatin regulation and HMT/DNMT activity in oligodendrocyte progenitor cells. This is the first work involving the study of *BHMT* expression in oligodendrocytes and where the authors suggest that *BHMT* is present in oligodendrocytes in both rats and humans, specifically in human MS tissue. They found that oxidative stress increases *BHMT* expression and showed that, in an oxidative environment, betaine can stimulate the expression of the oligodendrocyte maturation genes *SOX10* and *NKX-2.2*. Collectively, these data underscore that regulation of transcription factors required for oligodendrocyte differentiation by betaine could modulate oligodendrocyte maturation, likely impacting epigenetic changes including histone PTMs [74].

Another PTM of histones is citrullination, which represents the hydrolytic conversion from peptidyl-arginine to peptidyl-citrulline, catalyzed by enzymes belonging to the family of peptidylarginine deiminases (*PAD*). Citrullination plays an essential role in physiological processes, such as regulation of gene expression, apoptosis, and plasticity, while aberrant citrullination may be involved in the initiation and/or progression of autoimmune disorders such as MS. 

Mastronardi et al. looked at white matter from healthy subjects and NAWM tissue from MS brains to identify metabolic alterations that would make these areas more prone to eventual lesion development. To determine whether elevated nuclear *PAD-4* was accompanied by citrullination of nuclear proteins, they used a citrulline-specific monoclonal antibody (F95) to stain normal and MS white matter sections. They observed an increased *PAD4* with increased citrullination of histone H3 and TNF-α in MS NAWM and in the brains of animal models of demyelination. The mechanism involves nuclear translocation of PAD-4 [75]. This alteration may modify transcription and chromatin structure by altering the degree of methylation on arginine residues [76]. It is noteworthy that enzymatic inhibitors of this post-translational alteration can improve disease progression in mice models of demyelination, despite the fact that the functional relevance of histone citrullination has to be better understood.

Recently, Faigle et al., to better understand whether citrullination is implicated in distinct pathogenetic mechanisms in MS, i.e., whether structural proteins and/or those involved in inflammatory processes are citrullinated, with a proteomic approach, studied the role of citrullination in the brain tissue of patients affected by MS. However, based on their findings, they concluded that citrullination could be a side effect of an immunological or inflammatory response rather than having a key role in activating a T cell response [77]. 

Finally, there is emerging evidence that histone proteins post-translational modifications have roles in a number of fundamental MS-related processes (such as aging and neurodegeneration) and are susceptible to environmental variables. PTMs were suggested to be a mediator between oxidative stress, inflammation, and aging. Published data on PTMs in many age-related disorders, such as neurodegenerative diseases, provide evidence for a connection between histone PTMs and aging. Histone variations, DNA methylation, PTMs of histones, positioning of histones on the DNA sequence, and non-coding RNAs are some of the elements that contribute to the complicated process known as epigenome dysregulation that occurs with aging [78]. Future research should focus on intricate interactions between heritable and environmental factors that contribute to MS illness heterogeneity in order to better understand how histone PTMs variations affect these processes.

In conclusion, it is important to consider that histone modifications could act sequentially or in combination to form a recognizable “code” that increases the information of the genetic code [79,80], and decoding the complexity of histone marks is a challenge. Routinely antibody-based methods are used to investigate specific PTMs. However, these methods present some important limitations; for example, they are limited to confirming the existence of modifications and they are unable to identify unknown PTMs [81]. The mass spectrometry (MS) is progressively becoming an effective tool for studying histones. Recently, De Benedittis et al., using a top-down MS approach were able to quantify and identify, at the same time, several histone PTMs in the human lymphoblastoid cell line [82]. In the future, the development of technologies useful for a more in-depth and global screening of histone PTMs could have a profound impact on understanding their role in physiological and pathological processes.

## 4. miRNAs: Contribution in Patients with MS

Small ncRNAs, which have a broad ability to influence gene expression in conjunction with chromatin remodeling complexes, can also play a role in mediating epigenetic control. The ability of miRNAs to regulate the expression of hundreds of genes and consequently have an impact on a number of cellular processes makes them of particular interest among ncRNAs [83]. miRNAs are important participants in many biological processes and dysfunction of these molecules can affect the immune system, cause the release of inflammatory cytokines, and trigger the formation of autoantibodies, thus contributing to the pathogenesis of MS. It is becoming clear that miRNAs are essential regulators of both innate and adaptive immune responses, and their altered expression and/or function was linked to a variety of human diseases, including inflammatory conditions. The innate immune system is activated through toll-like receptors (TLRs), which by recognizing pathogen-associated molecular patterns, attract adapter proteins to the receptor and activate downstream signaling pathways. By governing the growth and activation of T and B cells, miRNAs were linked to adaptive immunity in addition to their function in innate immune system regulation [84]. It is well known that dysregulation or upregulation of proinflammatory/or neurotoxic miRNAs and/or downregulation of anti-inflammatory and neuroprotective miRNAs could contribute to MS onset and progression. An overview of the impact of different miRNAs and mediators on the pathophysiology of MS is presented in the work of Mohammed et al. [85].

Given that MS is the most prevalent chronic inflammatory and neurodegenerative disease, many research teams are looking for miRNAs that can predict the occurrence and course of the disease, and most of the studies examined miRNAs in immune tissues. However, altered miRNA profiles within both the central nervous system and the immune system were observed in the disease.

A comprehensive review examined the results of 61 papers investigating small non-coding RNAs in immune compartments and CNS tissue of MS patients, highlighting the most frequently dysregulated miRNAs across studies (dysregulated in at least four independent studies). miR-142-3p, miR-146a/b, miR-145, miR-155, miR-22, miR223/-3p, miR-326, and miR-584 were consistently found to be upregulated, while members of the miR-103, miR-15, miR-548, and let-7 families and miR-140 showed predominant downregulation in MS patients [86]. miRNAs dysregulation can result in altered levels of gene expression in many cell types involved in this disease. 

Most of the studies examined miRNAs in a case-control study, and used high-throughput miRNA profiling to identify dysregulated miRNAs. 

Furthermore, different RNA resources were used to study miRNAs expression, such as whole blood, as well as serum and PBMC, including lymphocytes [87]. The investigation of miRNA expression performed in 59 naïve MS patients (PPMS, SPMS, and RRMS) displayed that miR-17 and miR-20a were the most significantly different, and down regulated, in all MS groups vs. controls. Interestingly, gene expression analysis revealed that both miRNAs may regulate genes involved in T cell activation [88]. Investigating the expression of 365 miRNAs in lymphocyte, Lindberg et al., evidenced different miRNA expression profiles in CD4+, CD8+ T cells, and B cells of RRMS patients compared to controls. In particular, miR-17-5p was up-regulated in CD4+ cells of MS patients. The subsequent pathway analysis of potential miR-17 target genes further indicated that one of the most prevalent pathways was that of PI3K/Akt [89], which is known to be involved in lymphocyte development, activation, and survival [90,91]. In a subsequent study, a Taqman array of 667 miRNAs shed light on miRNA levels in subpopulations if not activated naïve CD4+ and in memory CD4+ T cells.

Comparing naïve T cells from MS patients to healthy controls, miR-128 and miR-27b are significantly higher in patients, while memory T cells show an increase in miR-340. Further, through the repression of their target genes, B lymphoma Mo-MLV insertion region 1 homolog (BMI1) and interleukin-4 (IL4), these miRNAs could induce inhibition of Th2 cell development and promote pro-inflammatory Th1 responses [92]. As it is known, a pro-inflammatory response is the main cause of the development of MS, where immune cells, such as T helper and cytotoxic T cells, B cells, and macrophages, infiltrate the central nervous system through the temporarily disrupted BBB. These cells, together with microglia and activated astrocytes, damage oligodendrocytes and myelin through the secretion of cytokines and chemokines that promote phagocytosis, ROS production, energetic crisis, and cytotoxic T cell activity, which then may cause myelin loss and or axon loss [93]. In the last ten years, several studies showed deregulated miRNA expression, both in blood cells and in CNS lesions, lineage-specific in particular cell populations, or at particular MS stages or subtypes. Further, during both relapse and remission periods, differential expression of various miRNAs was observed in PBMC of MS patients [94].

A better knowledge of the aetiology of multiple sclerosis as well as new alternative techniques for the diagnosis and treatment of the disease can be derived from the analysis of miRNA dysregulation and associated changes at the level of the miRNA/protein interaction [95]. Neurotrophic variables influencing neuroplasticity and neuroprotection in the CNS are known to be more important in neurodegenerative diseases such as MS.

When neuroprotective factors, such as nerve growth factor, brain-derived neurotrophic factor (*BDNF*), neurotrophin 3 (*NT-3*), and neurotrophin 4/5 (*NT-4/5*) are deficient in MS patients, the influx of PBMC cells to the CNS can make up for this, slowing the rate of cerebral atrophy. The protective impact on neuronal axons in the course of MS was linked to the *BDNF* factor released by PBMCs [91]. Heat shock proteins (HSPs) were demonstrated to have a neuroprotective effect by triggering anti-apoptotic pathways in addition to inhibiting the aggregation of misfolded proteins. The inflammatory process seen in the early stages of MS serves as a trigger for glial cells to express HSPs and release them into the extracellular environment, protecting nerve cells in the disease’s later neurodegenerative stages. Overexpression of most HSPs caused by inflammation and oxidative stress was seen in lesions in the CNS of MS patients and in an animal model of MS disease [96]. A class of histone deacetylases known as sirtuins (*SIRTs*) is controlled and dependent on the nicotinamide adenine dinucleotide. They participate in numerous processes that improve cell survival, while *SIRT1* preferentially inhibits genes linked to apoptosis and inflammation. In an animal model of MS, SIRT1 activation by SRT501 reduces brain dysfunction and stops neuronal death [97].

There is no information, however, on the function of miRNAs in controlling the level of expression of neuroprotective proteins, such as neurotrophies, heat shock proteins, and sirtuins, during the onset of multiple sclerosis.

In a recent work, Piotrzkowska et al., studied the effect of miRNAs on the expression levels of neuroprotective proteins, and some selected miRNAs. They specifically note a decrease in the expression of the *BDNF* and *SIRT1* genes, and an increase in the expression of miR-132-3p, miR-34a, and miR-132 in PBMCs, of MS patients, suggesting that the investigated miRNAs may regulate the expression-level genes under study. This drives the immune system towards the chronic inflammation seen in MS. The findings imply that the levels of selected neurotrophins (*BDNF* and *NT4/5*), heat shock proteins (*HSP70* and *HSP27*), *SIRT1*, and selected miRNAs in PBMC cells may function as biomarkers of inflammation in the CNS and neurodegenerative processes in MS patients. Investigations using animal models of neurodegenerative and demyelinating disorders demonstrated that *SIRT1* activation can slow the progression of the illness. *SIRT1* is a prospective novel target for MS treatment intervention as well as a potential biomarker of relapses. *SIRT1* modulation might be a useful strategy for MS treatment or prevention [98].

Dysregulation of miRNA expression and function may be related to MS diagnosis and pathogenesis, and miRNA-related pathways may also be used as a potential therapeutic target for MS. In the recent work of Erkal et at., they identify miRNA biomarkers for MS illness by in silico data analysis. They identify and analyze in detail four miRNAs (mir-212-3p, mir-98-5p, mir-142-3p, and mir-629-5p), and their target genes and related pathways were searched using different tools. For this investigation, immunological and neurological system-related pathways were given priority. They found that miR-142-3p, miR-98-5p, and miR-629-5p were upregulated and miR-212-3p was downregulated, and all were statistically significant. Their results demonstrate that miR-212-3p pathways (such as Toll-like receptor, neurotrophin signaling pathway, and cytokine–cytokine receptor interaction) and their common target genes *MAPK1*, *PIK3R3*, and *PIK3R1* have an effective role in the regulation of immune- and nervous-related systems. Compared to controls, they discovered that mir-98 has a greater expression level. According to their study, mir-98–5p is crucial for the JAK-STAT, ErbB, TGF-beta, and MAPK signaling pathways. Target genes of the mir-142-3p-associated pathways include *PIK3R5*, *PIK3CD*, *RAC1*, *AKT2*, and *PIK3R2*. Additionally, it was shown that these genes were connected to the PI3K-Akt signaling pathway. This pathway is involved in T cell responses. mir-142-3p, mir-98-5p, mir-629-5p, and mir-212-3p were shown to be statistically significant in the all-silico data analysis used to identify miRNA biomarkers for MS disease. Their target pathways and genes may also be useful in the pathophysiology of MS and aid in the diagnosis, prognosis, and therapy procedures [99]. Table 2 lists the studies discussed in the manuscript.

In conclusion, research assessed the miRNAs present in blood, biological fluids, or cells of MS patients, revealing specific miRNAs that differ between MS and healthy controls. While there is a lack of replication in the literature regarding miRNAs as biomarkers and conflicting results from various forms of research, several miRNAs were found with proven roles in inflammatory signaling, including regulation of lymphocyte subsets. Finally, some studies addressed the impact of therapy on miRNA expression by comparing naïve to treated patients [100]. However, collecting samples free from the effects of therapy is one of the challenges in MS research and the impact of therapy on miRNA expression that has to be take in account in biomarkers discovery.

## 5. Remarks on the Interaction of Epigenetic Changes

More and more evidence was given for epigenetic mechanisms being the bridge between genetics and environment in the pathogenesis of MS. The three epigenetic alterations (DNA methylation, histone modification, and miRNA regulation) that are currently best understood interact with one another. An increasing body of research demonstrates how these systems all interact. For instance, miRNA expression is controlled by DNA methylation [101], histone modification [102], and miRNAs themselves [103]. The recruitment of DNA methyltransferase 3A by histone modification, and connections between methylated DNA and histone deacetylases are two other interactions [104]. In summary, the evidence described here suggests that miRNAs and the epigenetic apparatus are both regulated in a reciprocal manner. Epigenetic regulation is carried out by miRNAs as part of the epigenetic machinery. The expression of miRNAs was epigenetically controlled by DNA methylations, and histone modifications. At promoter CpG islands, DNA methylation is frequently established, which limits the expression of miRNAs. Histone modification now has the power to promote and suppress miRNA translation activation. Enzymes involved in epigenetics can also be miRNA targets. By controlling the activity of epigenetics-related enzymes, miRNAs control how DNA methylation and histone modification are expressed. Additionally, it was discovered that some miRNAs are controlled by epigenetic regulators and, as a result, have an impact on the epigenetic machinery. Finally, these interactions imply that the epigenomic modifications are controlled not just by individual mechanisms, but also by intricate and poorly understood interactions between these fundamental mechanisms. However, given the possible significance of epigenetic modifications in MS, a deeper comprehension of these pathways and how they interact with other elements of the illness will be essential to comprehending and ultimately treating MS.

Further research in this area should occur soon given the growing array of tools available to manipulate and evaluate the effects of epigenetic modifications, such as oligonucleotide-modified miRNA profiles, histone modification via histone deacetylase inhibitors, and DNA methylation silencing via DNA methyltransferase inhibitors. Thus far, research demonstrated that autoimmune disorders possess a noteworthy epigenetic foundation, primarily attributable to the impact of environmental variables that modulate the immune system. Understanding the specific epigenetic modifications that take place in a patient’s genome can aid in determining which genes may require regulation and by what epigenetic regulatory pathways. Today, there is consensus that personalized epigenetics has the potential to transform the diagnostic, prognostic, and therapeutic approach for the treatment of autoimmune disorders such as MS. The interindividual differences in various epigenetic signatures, such as DNA methylation, histone modifications, and microRNAs, are the fundamental components of personalized epigenetics. Moreover, computational epigenetics opened up new avenues to use complex epigenomic data in patient diagnosis, prognosis, and treatment.

The potential application of epigenetic modifications and their regulation could definitely lead to the development of an entirely new field of methodology for the treatment and probable cure of MS.

## 6. Conclusions and Future Prospective

MS is a chronic condition that is influenced by both environmental and genetic factors. Despite the fact that the cause of the condition is unknown, epigenetic factors, such as DNA methylation and miRNA-based gene expression regulation, as well as histone modification, were linked to the pathogenesis of MS. Most MS-related genes are controlled by epigenetic processes in both immunological and central nervous system cells. Analysis of epigenetic modifications represents a useful strategy to decipher genome activity and its effect on the cellular and tissue dysfunction underlying MS. We summarized here the results of studies investigating epigenetic modifications. A large amount of research emphasized the significance of epigenetic mechanisms in the pathogenesis of MS, and studies so far made some progress in determining the involvement of epigenetics in MS. Several cellular processes including apoptosis, differentiation, and evolution can be modified along with epigenetic changes. Some alternations are associated with epigenetic mechanisms in MS patients and these changes can become key points for MS therapy. Indeed, knowledge of epigenetic modifications involved in the pathogenesis of MS opens a new avenue of research for identification of potential biomarkers, as well as finding new therapeutic targets. Although epigenetic therapy is still in its early stages, further research on the topic can be expected to reveal a new approach to understanding the phenotypic variations of MS. Drugs that can change abnormal histone modification, methylation, or miRNA expression in MS patients are the basis of epigenetic therapy [105].

Despite the lack of curative drugs capable of preventing the degeneration of nervous tissue, current therapies for MS are based on immunomodulatory and anti-inflammatory treatments. The goal of current MS treatments is to prevent further immune-mediated myelin degradation. Patients may still have progressive disability despite the widespread use of immunomodulatory treatments due to myelin regeneration failure and neuronal loss. Although these drugs reduce relapse rates, they fail to slow long-term disease progression of the disease or prevent neurodegeneration, and the long-term effects of early immunomodulatory intervention are not yet clear. Immunomodulatory drugs, such as interferons beta and glatiramer acetate, are used to treat MS; in particular, they reduce clinical attacks and the number of lesions [106]. However, these drugs can only be injected and have dangerous adverse effects. Furthermore, humanized monoclonal antibodies and oral drugs used to treat patients with MS exhibited several serious side effects, such as progressive multifocal leukoencephalopathy [107].

Patients with MS who are receiving therapy showed a variety of reactions. In order to direct these patients toward more efficacious treatments, predictive testing should be performed on them within the context of personalized medicine. In addition to the progress made in medication development, it is appropriate to select a therapy that considers each patient’s distinct genetic background. Thus, the discovery of genetic markers that impact the effectiveness of existing MS treatments can be viewed as a promising beginning that may lead to other discoveries down the road.

As epigenetic abnormalities, especially those that contribute to the etiology of autoimmune diseases, are better defined, it will be possible to find new epigenetic markers for early diagnosis and identification of new therapeutic targets. Importantly, epigenetic modifications are regulatory, stable, and reversible. Therefore, studying epigenetic mechanisms could represent a promising approach to better understand the mechanisms underlying MS and other diseases with similar complex etiology and improve therapeutic solutions. In conclusion, numerous studies established the importance of epigenetics in the pathogenesis of MS, and as a result, scientists are working to develop MS drugs with the fewest possible side effects. This can be accomplished by figuring out the specific mechanisms of epigenetics that contribute to MS pathogenesis. Further investigation into the epigenetic pathways underlying MS will undoubtedly open the door to creating novel diagnostic criteria, tracking disease activity, and designing epigenetically valid treatment strategies.

## Figures and Tables

**Table 1:** DNA methylation changes in MS patients.

Tissue	Cohort	Method	Findings	Reference
cell-free plasma DNA	RRMS in remission vs. HC; RRMS in exacerbation vs. HC; and RRMS in exacerbation vs. remission	microarray-based assay	RR-MS patients have an ICAM1 hypermethylation pattern in response to clinical remission, the observed differences mostly correlated with the disease process.	Ligget et al., 2010 [40]
Brain tissue	Matched NAWM and chronically demyelinated MS lesions	pyrosequencing	DNA methylation status in the promoter region of MBP, may be used to bidirectionally influence myelination and cellular differentiation in vitro.	Tiane et al., 2023 [41]
Blood	RRMS and SPMS vs. NNC	pyrosequencing	MBP, MAG, and CNTN2 showed a significant difference in methylation pattern between the control and MS sample.	Tiane et al., 2024 [42]
Buffy coat	69 MS (7 PPMS, 50 RRMS, 12 SPMS; 49 F, 20 M), 19 HC	cloning BS-sequencing	Increased DNA methylation level of SHP-1 promoter 2 in MS compared with HC. No relationships between DNA methylation and MS clinical parameters (EDSS, disease duration, phase).	Kumagai et al., 2012 [45]
CD4+ T cells	17 RRMS (10 MS tysabri, 3 glatiramer acetate, 2 IFN-1b treated, 2 nontreated), 7 HC	cloning BS-sequencing	No difference in DNA methylation level of FOX3P and IL-17A between MS tysabri-treated patients and HC. Demethylation of FOX3P and IL-17A loci in tysabri untreated patients compared with HC.	Janson et al., 2011 [46]
Brain NAWM	DC: 28 MS; VC: 10 MS, 20 NNC	Illumina 450 K array	220 hypomethylated DMRs (1235 CpGs) and 319 hypermethylated DMRs (1292 CpGs) at oligodendrocyte specific genes (BCL2L2, HAGHL, NDRG1, CTSZ, andLGMN). Correlation with expression of a portion of corresponding genes.	Huynh et al., 2014 [51]
Hippocampus	myelinated (n = 8) or demyelinated MS (n = 7)	Illumina 450 K array	Hyper-methylation in 10 genes and hypo-methylation in 6 genes.	Chomyk et al., 2017 [52]
Brain tissue	active lesion, chronic active lesion, chronic inactive lesion and NAWM vs. HC	Illumina 450 K array	Decreased activity of the transcription factor CREB, linked to neuro-axonal dysfunction in MS patients vs. controls.	Kular et al., 2019 [53]
Brain tissue	active lesion, chronic active lesion, chronic inactive lesion and NAWM vs. HC	Illumina 450 K array	A total of 1226 significantly differentiably methylated sites between MS and NNC.	Kular et al., 2022 [54]
CD14+ monocytes	PPMS vs. HC	Illumina 450 K array	A total of 169 DMPs, 90.5% of which were hypermethylated in PPMS patients.	Kiselev et al., 2020 [55]
CD4+ and CD8+ T cells, CD14+ monocytes and CD19+ B cells	RRMS, SPMS and HC	EWAS	A total of 1511, 666, and 30 significant DMPs in CD19+, CD14+, and CD8+ cells, respectively, between RRMS, SPMS, and HC.	Ewing et al., 2019 [58]
CD4+ T lymphocytes and CD14+ monocytes	treatment-naïve RR-MS patients and HC	EWAS	In CD4+ cells of MS patients, most of the DMPs were hypomethylated, while in CD14+ cells they were hypermethylated.	Kiselev et al., 2022 [59]
CD4+ T cells	30 RRMS (interferons, glatiramer acetate, natalizumab or fingolimod treated), 28 HC	Illumina 450 K array	Correlation of HLA-DRB1 DNA methylation status and HLA-DRB1*15:01 haplotype. Differently methylated CpG sites (19) inside of MHC region and 55 outside.	Graves et al., 2014 [60]
Blood,	MS vs. HC	EWAS	Negative correlation between gene expression, and methylation at DMR-2	Xavier et al., 2023 [62].

HC, healthy controls; RRMS, relapsing-remitting multiple sclerosis; SPMS, secondary progressive multiple sclerosis; PPMS, primary progressive multiple sclerosis; NNC, nonneurological controls; EDSS, expanded disability status scale; DC, discovery cohort; VC, validation cohort; IFN-1b, interferon beta-1b; NAWM, normal appearing white matter; BS, bisulfite; MBP, myelin basic protein gene; DMRs, Differentially methylated regions; DMPs, differentially methylated positions; and EWAS, epigenome-wide association study.

**Table 2:** Studies that detailed alterations in miRNA expression levels associated with multiple sclerosis.

Sources	Methodology	Cohort	Upregulated miRNAs	Downregulated miRNAs	Reference
N.A.	comprehensive literature review (61 papers)	N.A.	miR-142-3p, miR-146a/b, miR-145, miR-155, miR-22, miR223/-3p, miR-326, miR-584	miR-103, miR-15, miR-548, let-7, miR-140	Piket et al., 2019 [86]
Whole blood	microarray analysis	59 treatment naïve MS patients (18 PPMS, 17 SPMS, 24 RRMS) vs. 37 HC		miR-17 and miR-20a	COX et al., 2010 [88]
CD4^+^	qRT-PCR	8 RRMS patients vs. 10 HC (discovery cohort) 23 RRMS patients vs. 20 HC (validation cohort)	miR-485-3p, miR-376a, miR-497, miR-193a, miR-126, miR-17-5p	miR-34a	Lindberg et al., 2010 [89]
naïve CD4+ T cells, memory CD4+ T cells	qRT-PCR	22 treatment naïve MS patients (5 PPMS, 5 SPMS, 12 RRMS) vs. 16 HC	miR-128, miR-27b (naïve CD4+ T cells), miR-340 (memory CD4+ T cells)		Guerau-de-Arellano et al., 2011 [92]
PBMC	qRT-PCR	28 MS patients (15 SPMS, 13 RRMS) vs. 33 HC	miR-132, miR-21-5p, miR-181b-5p, miR-34a, miR-132-3p	miR-134	Piotrzkowska et al., 2021 [98]
PBMC	Datasets analysis (from GEO)	66 MS patients vs. 185 HC	mir-142-3p, mir-98, mir-629	hsa-mir-212	Erkal et al., 2022 [99]

N.A., not applicable; GEO, NCBI Gene Expression Omnibus; HC, healthy controls; RRMS, relapsing-remitting multiple sclerosis; SPMS, secondary progressive multiple sclerosis; and PPMS, primary progressive multiple sclerosis.
